# The Accuracy of Computerized Adaptive Testing in Heterogeneous Populations: A Mixture Item-Response Theory Analysis

**DOI:** 10.1371/journal.pone.0150563

**Published:** 2016-03-01

**Authors:** Richard Sawatzky, Pamela A. Ratner, Jacek A. Kopec, Amery D. Wu, Bruno D. Zumbo

**Affiliations:** 1 School of Nursing, Trinity Western University, Langley, British Columbia, Canada; 2 Centre for Health Evaluation and Outcomes Science, Providence Health Care Research Institute, Vancouver, British Columbia, Canada; 3 Faculty of Education, University of British Columbia, Vancouver, British Columbia, Canada; 4 School of Population and Public Health, University of British Columbia, Vancouver, British Columbia, Canada; 5 Arthritis Research Centre of Canada, Vancouver, British Columbia, Canada; 6 Measurement, Evaluation & Research Methodology, University of British Columbia, Vancouver, British Columbia, Canada; 7 University of British Columbia, Vancouver, British Columbia, Canada; University of East Piedmont, ITALY

## Abstract

**Background:**

Computerized adaptive testing (CAT) utilizes latent variable measurement model parameters that are typically assumed to be equivalently applicable to all people. Biased latent variable scores may be obtained in samples that are heterogeneous with respect to a specified measurement model. We examined the implications of sample heterogeneity with respect to CAT-predicted patient-reported outcomes (PRO) scores for the measurement of pain.

**Methods:**

A latent variable mixture modeling (LVMM) analysis was conducted using data collected from a heterogeneous sample of people in British Columbia, Canada, who were administered the 36 pain domain items of the CAT-5D-QOL. The fitted LVMM was then used to produce data for a simulation analysis. We evaluated bias by comparing the referent PRO scores of the LVMM with PRO scores predicted by a “conventional” CAT (ignoring heterogeneity) and a LVMM-based “mixture” CAT (accommodating heterogeneity).

**Results:**

The LVMM analysis indicated support for three latent classes with class proportions of 0.25, 0.30 and 0.45, which suggests that the sample was heterogeneous. The simulation analyses revealed differences between the referent PRO scores and the PRO scores produced by the “conventional” CAT. The “mixture” CAT produced PRO scores that were nearly equivalent to the referent scores.

**Conclusion:**

Bias in PRO scores based on latent variable models may result when population heterogeneity is ignored. Improved accuracy could be obtained by using CATs that are parameterized using LVMM.

## Introduction

Computerized adaptive tests (CATs) increasingly are used to quantify health-related concepts, including patient reported outcomes (PROs) pertaining to symptoms, functional status, and mental health [1–8]. CATs are computerized systems that involve the selective administration of measurement items (questions) from a large bank of items for the measurement of a common construct (e.g., a PRO). The selection of items differs for each individual and is based on the individual’s responses to prior items (i.e., it is adaptive to emerging information about the individual’s level on the measured construct). This latent variable model-based approach to measurement applies item response theory (IRT) to estimate measurement model parameters that are subsequently used to determine individuals’ scores on a latent variable based on their responses to multiple questions or items. The advantage of using CATs for the determination of individuals’ PRO scores is that they can minimize response burden by selectively administering those items that are most likely to be relevant to an individual’s health status. With their application, the most informative measurement at a desired level of precision can be obtained with efficiency [9]. Relative to other measurement approaches that require the administration of a full, fixed set of items to all people, such as the use of summed scores derived from validated questionnaires, CATs can be shorter, uniquely targeted to an individual’s status, and more accurate [10–13].

The advantages of CATs, derived from the theoretical foundations of IRT [14], result from the principle of local independence. Most commonly, a unidimensional IRT measurement model is used to specify the relationships between measurement items and the measured construct. In the case of a unidimensional IRT model, local independence implies both *item* homogeneity and *sample* homogeneity [15, 16]. Item homogeneity refers to the exchangeability of items from the same item bank, which is necessary to warrant the selective administration of different items to different people, as is the case in a CAT. If item homogeneity holds, different combinations or sets of items can be used to measure the same construct. Sample homogeneity refers to the exchangeability of sampling units (people or groups), and is necessary to ensure that the scores of different people are comparable. If the condition of sample homogeneity is not met, it is impossible to determine the extent to which observed between-subject differences in the model-predicted scores reflect actual differences in the PRO being measured, or whether they are due to other factors that may influence individuals’ responses to the items.

Researchers have revealed that people’s responses to PRO measures may be influenced by differences in their age, gender, bodyweight, ethnicity, or other factors [17–19]. Typically, research related to these potential sources of heterogeneity in measurement employs some form of differential item functioning methods [20–25]. These methods can only be applied when potentially relevant group differences can be determined *a priori* and when empirical data on these characteristics are available. However, it is possible that there are unknown or unmeasured characteristics within a sample, or interactions among such characteristics, that may influence individuals’ responses to items [26–29]. Accordingly, researchers have recommended the use of latent variable mixture models (LVMM) to examine the possibility of heterogeneity in a sample with the measurement of a construct [26–28, 30–33]. These mixture models, including factor-mixture models for continuous items and IRT- or Rasch-mixture models for categorical items, are specified by allowing the measurement model parameters to vary across two or more latent classes (i.e., hidden subpopulations of people) [34]. If latent classes of people are identified in the mixture model, then the sample of individuals is said to be heterogeneous with respect to the measurement model. That is, a single set of measurement model parameters will not be equivalently applicable to all of the individuals in the sample because their responses are moderated by factors other than the PRO being measured. The estimated measurement model parameters will be biased if such heterogeneity in the sample is not accommodated. This bias will affect the accuracy and trustworthiness of the measurement model-predicted PRO scores, including those produced by a CAT.

We previously demonstrated the implications of unaddressed sample heterogeneity with respect to a fixed-length PRO measure consisting of 10 items measuring physical functioning [34]. We reported that failure to meet the condition of sample homogeneity led to inconsistencies in the reliability of the measurement items across sample subgroups, bias in the model predicted PRO scores of a substantial proportion of the sample, and poorer measurement precision, particularly in the tails of the score distribution. We also demonstrated how LVMM, in conjunction with IRT, could be used to obtain improved model-predicted PRO scores that accommodate such sample heterogeneity. However, in our earlier work, we did not explore the implications of sample heterogeneity with respect to the application of a CAT using a larger item bank. We also did not explore whether a CAT programmed with a LVMM (instead of a conventional IRT model) would produce more accurate PRO scores.

We describe, herein, a LVMM analysis and simulation study for which we used prior model-based parameters of an item bank for the measurement of pain (i.e., the calibrated items used in a CAT). Our analytical objectives, using the measurement of pain as an example, were to (a) examine the implications of sample heterogeneity with respect to latent variable model-predicted PRO scores and (b) determine the extent to which a CAT programmed with a LVMM would produce improved accuracy in the prediction of PRO scores (relative to a CAT programmed with a conventional IRT model, ignoring sample heterogeneity). We achieved these objectives by comparing the results of the following four models, all of which were unidimensional:

1. a measurement model of *all* items in the item bank assuming sample homogeneity,
2. a latent-variable mixture model of *all* items in the item bank (i.e., accommodating sample heterogeneity),
3. a CAT that applied a single set of measurement model parameters to all people (i.e., ignoring sample heterogeneity), and
4. a CAT that applied a latent-variable mixture model (i.e., accommodating sample heterogeneity).

## Methods

The methods section is organized in three parts: (a) we first describe the instrument and sample that provided the observed data, (b) we describe how the model-based parameters were established, and (c) we then describe the simulation methods used to examine the implications of sample heterogeneity and the use of CAT.

### The observed data

The CAT-5D-QOL [35] consists of five domains that are relevant to people with joint problems (i.e., pain or discomfort, daily activities, walking, handling objects, and feelings). The item banks, one for each of the five domains, were developed by selecting items from a pool of 1,400 candidate items taken from various published instruments or generated from open-ended interviews with people with arthritis. The instrument has undergone extensive evaluation. The developers of the CAT-5D-QOL administered the 219 selected items to a “calibration” sample of individuals with physician-diagnosed or self-reported arthritis. Factor analysis was used to examine the unidimensionality of the item banks, and measurement invariance was assessed using IRT-based techniques to compare item parameters across groups characterized by differences in gender and age. The reliability and validity of the CAT-5D-QOL was further examined in a sample of adults with back pain [36]. The results of this validation study demonstrated satisfactory reliability estimates for the five domains (ranging from .83 to .92), and expected patterns of correlations with established instruments including the SF-36 Health Survey [37].

In this report, we limit our attention to one of the five CAT-5D-QOL domains, the Pain item bank, which consists of 36 items measuring the severity and frequency of pain or discomfort and the impact of pain on activities of daily living and leisure. The response options varied for the items: 17 items had responses ranging from 1 to 5 for “not at all” to “extremely”, 12 items had responses ranging from 1 to 5 for “never” to “always”, and 7 items had a range of item-specific response options.

The items had been administered to a heterogeneous sample of 340 adults who attended two rheumatology clinics in the City of Vancouver, Canada, 331 adults with osteoarthritis who were awaiting joint replacement surgery in the province of British Columbia, Canada (i.e., they were waitlisted), and 995 randomly sampled community-dwelling adults, drawn from a telephone directory, who participated in a mailed survey (21.8% had rheumatoid arthritis or osteoarthritis). The response rates for the three sampling frames were 60%, 72%, and 33% corresponding to the rheumatology clinic, the joint replacement waiting list, and stratified-random community samples, respectively [35]. Because this was a low risk descriptive survey study, signed consent was not required for participation. All participants were provided a consent form together with the survey questionnaire and were informed that their consent was implied if they completed the questionnaire. The study and consent procedures were approved by the University of British Columbia, Behavioural Research Ethics Board (approval: B00-0500).

### Establishing model-based parameters and producing PRO scores

As a necessary first step to confirm that the 36 pain items arose from one and only one factor (i.e., the condition of unidimensionality was met), we conducted an exploratory factor analysis of the polychoric correlations using a GEOMIN rotation, which addressed the ordinality of the data [38]. We examined the eigenvalues, a scree plot, and the residual correlations to identify the best solution.

We then applied an IRT-based latent variable mixture modeling (LVMM) approach, using the M*plus* [39] software, to estimate item parameters that accommodated sample heterogeneity. We followed the LVMM specifications described in detail by Sawatzky et al. [34]. This unidimensional mixture model specified latent classes that divided the sample into relatively homogeneous classes or subgroups, which allowed for class-specific estimation of the measurement-model parameters [40–42]. The LVMM that we applied was a mixture of the Samejima [43] two-parameter logistic graded response model. This model, which has been shown to be equivalent to an ordinal-data factor model [44–46], specified the relationships between the continuous latent factor for Pain (the model predicted PRO scores) and the 36 ordinal indicators with a proportional odds logistic link function. As is conventional in IRT and for the purpose of model identification, the distribution of the latent factor (the model predicted PRO scores) was scaled with a variance of 1.0 and a mean of 0.0. Only the measurement model parameters, including the slopes (analogous to factor loadings or discrimination parameters) and thresholds (from which difficulty parameters are derived in IRT), were allowed to vary across the latent classes. In so doing, any found latent classes would be defined by a lack of invariance in the measurement model parameters. A robust maximum likelihood estimator (MLR) was applied to estimate the model parameters using the M*plus* software [39] (see Sawatzky et al. [34] for further details).

To determine the appropriate number of classes we compared the relative fit of models specified with one, two, or three latent classes. We evaluated the univariate model fit (i.e., the difference between the predicted and observed item responses of the sample) and the bivariate model fit (i.e., the joint distributions of the predicted and observed item responses) of these three models by examining the standardized difference (residual) scores [47, 48]. Chi-square tests were employed to facilitate these assessments. We also compared relative fit indices (i.e., the Bayesian information criterion (BIC) [49], the Vuong-Lo-Mendell-Rubin Likelihood Ratio Test (VLMRLRT) [50–52], the bootstrapped likelihood ratio test (BLRT) [53, 54], and the entropy values of the three models. The model with the smallest BIC, a statistically significant VLMRLRT and BLRT, and a satisfactory entropy value (i.e., ≥ .80) was selected as the best fitting [55]. Latent class membership was determined using the latent class posterior probabilities [10, 56]. The LVMM parameter estimates are referred herein as the referent parameter estimates and the corresponding saved model predicted PRO scores are the referent scores.

Logistic regression differential item functioning (DIF) techniques were used to describe the magnitude of the differences in the measurement model parameters across the latent classes [22, 25]. This involved a two-step ordinal logistic regression analysis of each item’s distribution (the observed data). The first step regressed the item on latent class membership and the model predicted PRO scores of the conventional IRT model to evaluate uniform DIF (i.e., differences that are constant across the range of predicted scores). The second step regressed the item on latent class membership, the model predicted PRO scores, and their interaction to examine non-uniform DIF (differences among the latent classes that depended on the predicted scores). We evaluated the magnitude of the differences based on changes in the items’ pseudo *R-*squares (> .035 was considered moderate and > .070 was large) [25, 57]. In addition, we used multinomial logistic regression analysis with pseudo-class draws to describe differences in health-related and demographic variables across the latent classes and to predict class membership [58–60].

### Monte-Carlo simulation methods

**Fig 1:**
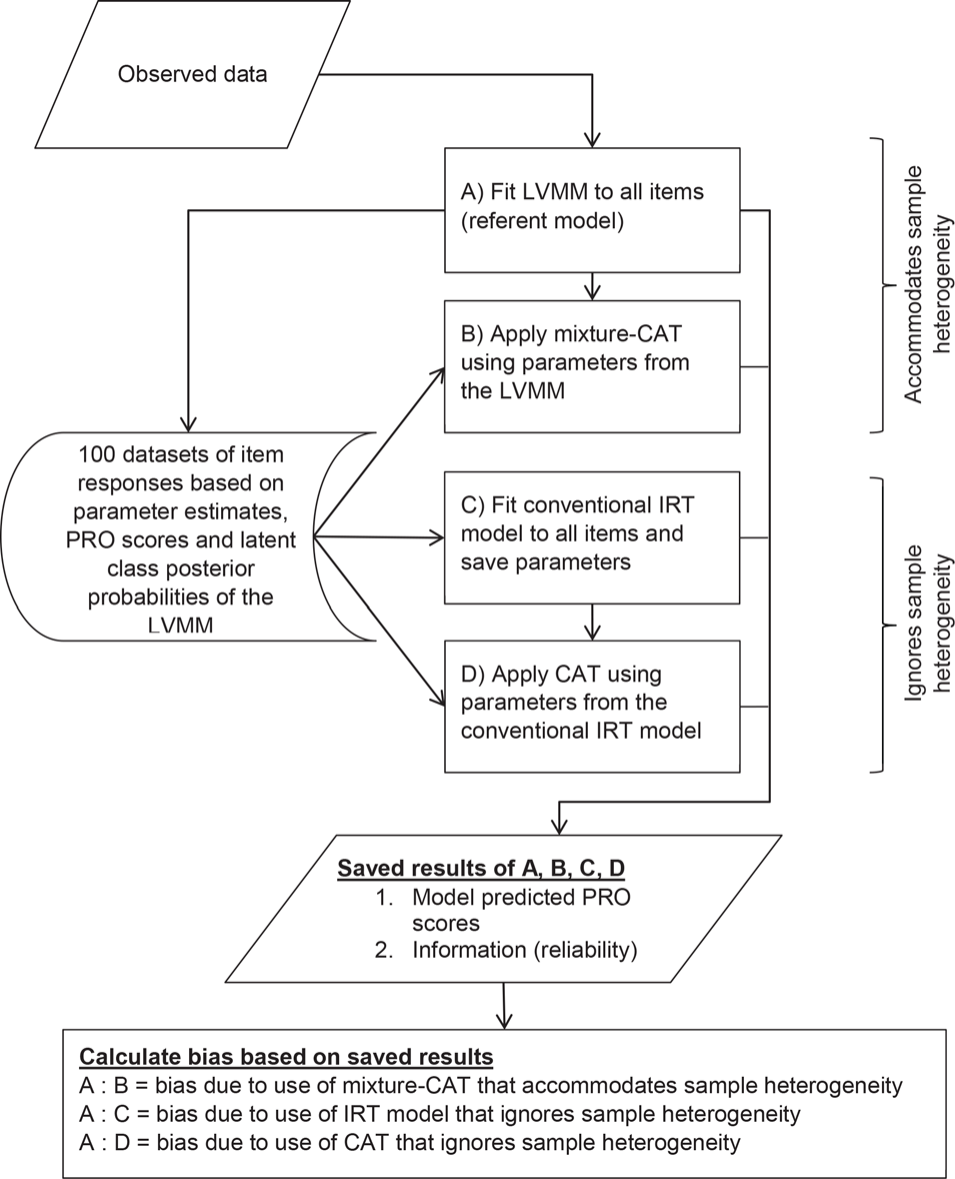
Flowchart of simulation analyses.

The referent parameter estimates and model predicted PRO scores of the best fitting LVMM of the pain subscale items were used as the basis for the Monte-Carlo simulation. This involved four steps which are outlined in Fig 1. All four steps produced a set of predicted PRO scores and corresponding information (reliability). The referent parameter estimates and model predicted PRO scores derived in the first step (i.e., step “A” in Fig 1), which were based on the LVMM described above, were used to generate 100 datasets each with 10,000 fictitious respondents. The process for generating the data was as follows: (a) randomly select 1,000 respondents from the original dataset; (b) following the procedures described by Sawatzky et al. [34], use the within-class PRO scores, item parameters, and the posterior probability of latent class membership to compute the cumulative probability for each item response category; (c) follow the approach of Hambleton and Rovinelli [61], as applied to ordinal data by Koh [62], to generate item scores by first multiplying each cumulative probability with a random value from a normal distribution and subsequently selecting the item category with the highest value. Thus the generated datasets were intentionally heterogeneous and reflective of the original sample distributions in that they were based on measurement model parameters of multiple latent classes and the corresponding model predicted PRO scores based on the original sample of observed data.

As the second step (step “B” in Fig 1), a “mixture-CAT” that used the reference class-specific parameter estimates (i.e., based on the LVMM) was applied to the 100 datasets. The latent-variable model-predicted PRO scores resulting from the “mixture-CAT” accommodated sample heterogeneity by adding each class-specific PRO score weighted by its corresponding posterior probability of latent class membership (please refer to Sawatzky et al. [34] for detailed information about the computations). The information was similarly weighted. Thus, the sequential steps of the CAT were as follows: (a) compute the three within-class PRO scores and information based on the item responses, (b) obtain the weighted PRO scores and information (reliability) by adding the within-class values multiplied by the corresponding posterior probabilities used to generate the data, and (c) determine whether the stopping rules have been met and, if not, administer the next item with the greatest information. We saved the PRO scores of the mixture-CAT and the information (reliability) that were achieved when the stopping rules described below were reached. The PRO scores were then averaged across the 100 datasets.

The third step (step “C” in Fig 1) involved fitting a conventional IRT model, which ignored sample heterogeneity, for each of the 100 generated datasets. We used a unidimensional graded response IRT model (without any latent classes). The fourth step (step “D” in Fig 1) was to apply a “conventional CAT”, which was based on the parameters estimated in the third step (i.e., ignoring sample heterogeneity), to each of the 100 datasets. Thus the PRO scores from steps three and four, and which were averaged across the 100 datasets, did not accommodate sample heterogeneity.

All CATs (both the conventional and mixed) started with the same item. Subsequent items were selected following established CAT methods. That is, the next item was selected by identifying which of the non-administered items would contribute the greatest information at the current value of the model predicted PRO score. There are many suggestions for stopping rules based on the desired level of precision and the maximum number of items to be administered [14, 63]. Herein, we report the results based on a relatively conservative standard error threshold of 0.20 with the goal of achieving high reliability estimates. There are instances at the tails of the distributions where a standard error of 0.2 would never be achieved. Considering that a goal of CAT is to administer relatively few items, we therefore added a second stopping rule to administer no more than 10 items if the first condition was not met. Taken together, these stopping rules produced a high average reliability estimate of 0.96; a reliability estimate of greater than 0.90 was achieved for 92% of the sample, and none of the sample had a reliability estimate of less than 0.70.

At the completion of the above four steps, we evaluated the extent of bias in the computation of model-predicted scores resulting from the use of a conventional IRT model with invariant measurement model parameters (i.e., ignoring sample heterogeneity). There were three sets of results that were compared to those of the referent results of the LVMM (results of A in Fig 1), including: (A:B) the “mixture-CAT” (using parameters from the LVMM that accommodated sample heterogeneity), (A:C) the conventional IRT model (including all 36 items) and (A:D) the corresponding “conventional CAT.” Bias was evaluated with respect to the model-predicted PRO scores (which were scaled with a mean of zero and a variance of one).

## Results

Distributions of demographic and health-related variables in the sample are provided in Table 1. The exploratory factor analysis of the observed data (i.e., responses to the 36 items) revealed a large dominant factor with an eigenvalue of 27.0, which was 14.2 times greater than the second eigenvalue of 1.9. The factor loadings ranged from .75 to .96. A single-factor confirmatory factor analysis with weighted least squares estimation using polychoric correlations resulted in a RMSEA of .046 and a CFI of 1.000, which were indicative of a very well-fitting model [64–66]. The model predicted PRO scores closely approximated a normal distribution (skew = 0.08 and kurtosis = 0.73). Having demonstrated item homogeneity, we proceeded to fit a unidimensional IRT model and to examine sample heterogeneity.

**Table 1:** Description of the Sample and Latent Classes.

	Prevalence				Multivariate logistic regression^b^		
Variables	Full sample	Class 1^a^	Class 2^a^	Class 3^a^	OR (95% CI) classes 1 versus 3	OR (95% CI) classes 2 versus 3	OR (95% CI) classes 2 versus 1
Sex (referent = male)	60.6	63.3	63.2	57.1	1.1(0.8;1.4)	1.0(0.8;1.5)	1.1(0.8;1.4)
Age (mean (sd))^c^	57(15.9)	58.3(17.5)	56.9(16.0)	55.3(17.3)	1.0(0.9;1.1)	0.9(0.8;1.0)	0.9(0.8;1.0)
Taking medications	77.9	85.8	84.2	67.9	1.8(1.2;2.7)	1.8(1.3;2.7)	1.0(0.6;1.7)
Hospitalized during past year	20.5	27.2	19.2	17.3	1.2(0.9;1.7)	0.8(0.6;1.2)	0.7(0.5;1.0)
Has rheumatoid arthritis	28.0	37.4	27.9	21.9	1.2(0.7;2.0)	1.4(0.9;2.3)	1.2(0.7;2.1)
Has osteoarthritis	36.6	40.7	45.5	27.9	1.5(1.0;2.0)	2.1(1.4;3.0)	1.4(1.0;2.1)
Has another health condition	77.3	81.1	83.6	70.6	1.3(0.9;1.8)	1.5(1.1;2.2)	1.2(0.8;1.9)
Self-reported health is fair or poor (referent = good, very good or excellent)	24.0	32.7	27.0	16.5	1.6(1.1;2.3)	1.5(1.1;2.1)	0.9(0.7;1.3)
Sampling groups							
Community-dwelling (referent)	59.8	48.7	55.8	67.5	1.0	1.0	1.0
Rheumatology clinic sample	20.4	29.7	16.7	15.7	1.4(0.8;2.5)	0.6(0.3;1.0)	0.4(0.2;0.7)
Awaiting joint replacement surgery sample	19.8	21.6	24.5	16.8	0.9(0.6;1.5)	0.8(0.5;1.2)	0.8(0.5;1.4)

*Notes*. *OR* = odds ratio. N = 1,660

^a^ Prevalence computed based on posterior-probability based multiple imputations using the M*plus* software. Proportions of latent class membership are .27, 30 and .43 for classes 1, 2 and 3, respectively.

^b^ Odds ratios based on the multinomial logistic regression using pseudo-class draws.

^c^ For each 10-year (decade) increase in age.

The comparative fit of the one-class unidimensional IRT model (which ignored sample heterogeneity) with that of 2- and 3-class LVMMs revealed that the sample was heterogeneous with respect to the unidimensional IRT model (the sample was not large enough to fit a 4-class model). The 3-class LVMM was the most defensible model that provided significant improvement in model fit relative to the 1- and 2- class models (i.e., it produced the smallest Bayesian Information Criterion, a statistically significant bootstrapped likelihood ratio test, and entropy of .83; see Table 2 for comparative fit indices). The model predicted PRO scores were distributed with a mean of zero and a standard deviation of .97 in the overall sample (means and standard deviations were .10 (.93) in class 1, -.13 (1.01) in class 2, and .12 (.90) in class 3), and were relatively normally distributed with skewness and kurtosis values of .03 and -.53, respectively (the corresponding class-specific values were: .14 and -.01 in class 1, .05 and -.81 in class 2, and .04 and -.71 in class 3). Only 4% of the respondents had no pain at all (i.e., they provided the response indicative of no pain for all 36 items). All of the class-specific slopes were statistically significant and of substantial magnitude; the smallest standardized slope was .76 and the median value of all slopes across the three classes was .89. The item parameter estimates are provided in the S1 Appendix.

**Table 2:** IRT Mixture Analyses of the Pain Item Bank.

								Class proportions*		
*K*	*P*	LL	BIC	LR	VLMR p-value	BLRT p-value	Entropy	C1	C2	C3
1	177	-43285.9	87884					1.00		
2	354	-41715.4	86056	3141	.000	.000	.86	.59	.41	
3	531	-40858.6	85654	1713	.000	.000	.83	.27	.30	.43

*Notes*. *N* = 1,660. *K* = Number of latent classes in the model. *P* = number of parameters. LL = log likelihood. BIC = Bayesian Information Criterion. LR = Likelihood ratio of GRM and 2-, and 3-class LVMMs. VLMR = Vuong-Lo-Mendel-Rubin likelihood ratio test *p*-value. BLRT = Bootstrapped likelihood ratio test *p*-value. C1-C3 = classes 1 through 3.

* Probability of latent class membership predicted by the model.

**Fig 2:**
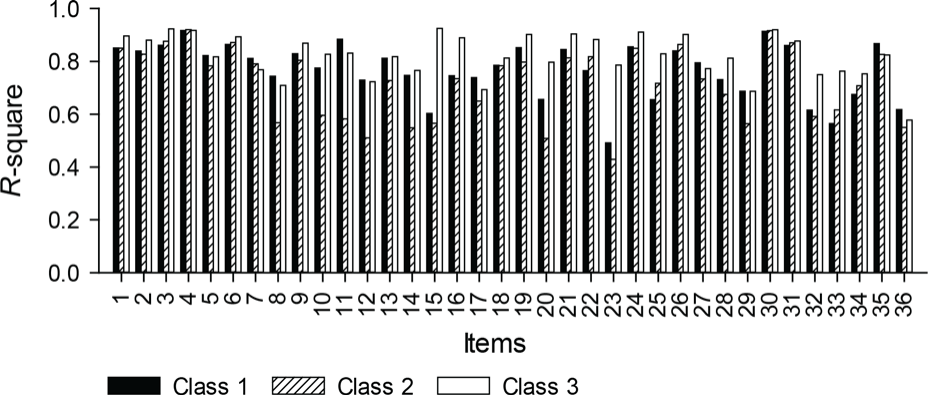
Explained within-class item-variances of the LVMM.

In examining the magnitude of the differences in the class-specific slopes, we noted that, of the 36 items, 16 had statistically significant uniform DIF and 9 had non-uniform DIF (*p <* .001). The within-class item variances in Fig 2 provide a representation of the relative importance of items to the model-predicted scores. Seven of the items were identified as having moderate DIF (.035 ≤ Δ*R*^*2*^ ≤ .070) and three as having large DIF (Δ*R*^*2*^ > .070). Examples of the least invariant items included: (item 23) “During the past 4 weeks, how often did pain prevent you from eating your meals?”, which had lower item variances in classes 1 (*R*^*2*^ = .49) and 2 (*R*^*2*^ = .43), relative to class 3 (*R*^*2*^ = .79); and (item 11) During the past 4 weeks, how much did pain or discomfort interfere with your self-care activities, such as dressing or bathing?”, which had lower items variances in class 2 (*R*^*2*^ = .58), relative to classes 1 (*R*^*2*^ = .88) and 3 (*R*^*2*^ = .83). In other words, interference of pain with eating was of greater importance to the model-predicted scores in class 3, and interference of pain with self-care activities was of lesser importance in class 2. Examples of the most invariant items include: (item 4) “how much did pain or discomfort interfere with your normal work or other daily activities?” and (item 5) “how much did pain or discomfort affect your ability to fall asleep?”

Latent class membership was distributed with 25%, 30%, and 45% of the respondents assigned to classes 1, 2 and 3, respectively. Class membership was partially explained by the three sampling groups and several of the measured demographic and health-related variables in our study. That is, consistent with the notion of latent classes, there are other unknown or unmeasured characteristics within the sample, and interactions among them, that further explain latent class members. Nevertheless, the multivariate multinomial logistic regression analysis of class membership revealed that, with respect to the measured variables in our study, relative to people in class 3, people in classes 1 and 2 were more likely to be taking medications and to report having fair or poor health (see Table 1). People in class 2, relative to those in class 3, were more likely to have osteoarthritis, to have a health condition other than osteoarthritis or rheumatoid arthritis, and to be from the community-dwelling sample. Classes 1 and 2 were fairly similar with the exception that class 2 members were less likely to have been hospitalized and from the rheumatology clinic sample.

### Simulation analyses

**Fig 3:**
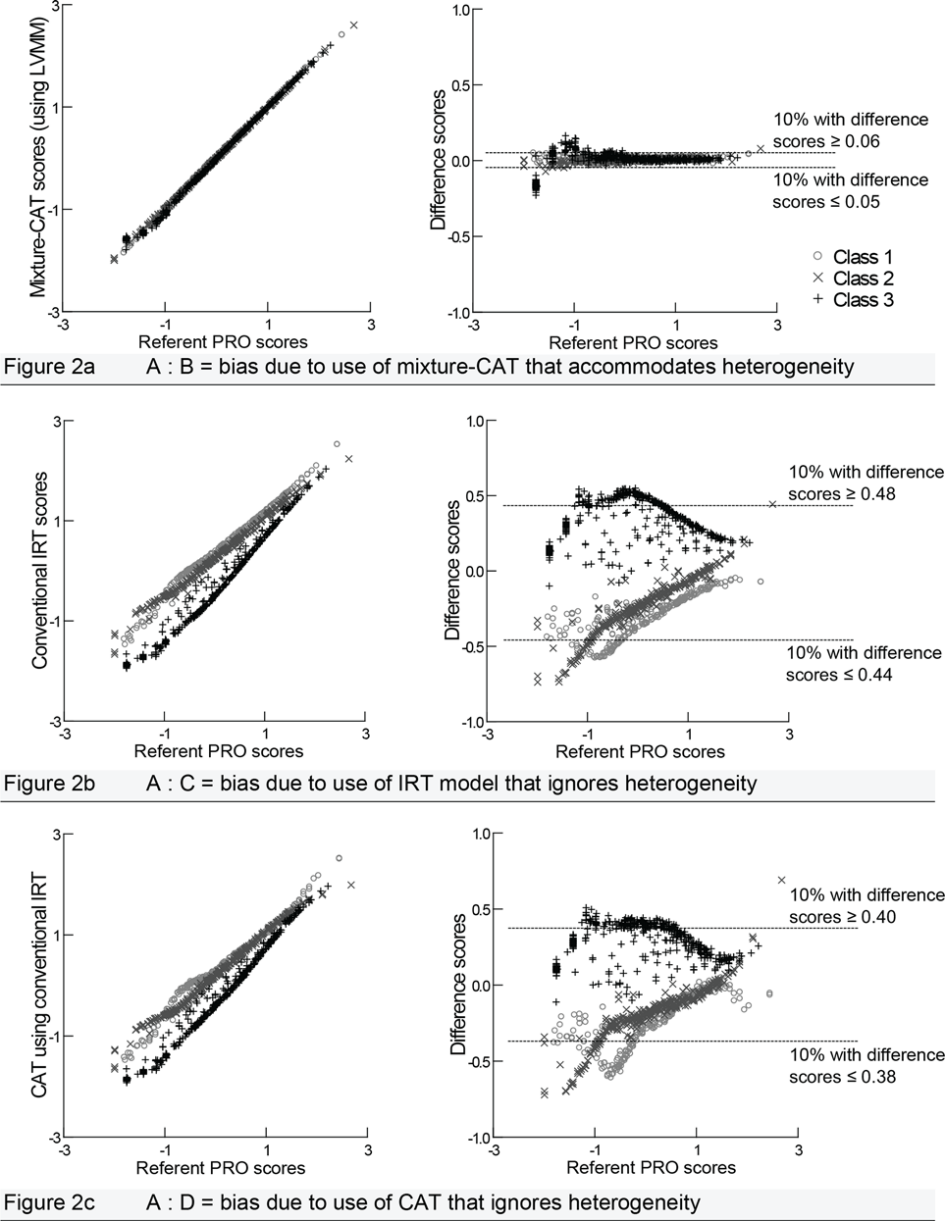
Impact of sample heterogeneity with respect to the predicted scores. Differences scores are the referent PRO scores minus the model predicted PRO scores based on 1,000 observations averaged across 100 simulated datasets. Although these are not class-specific scores (the referent scores are based on the LVMM), the latent classes are superimposed, as determined by the largest posterior probability, to visualize the bias within each class.

The number of items administered by the mixture-CAT ranged from 3 to 10. On average, 49% of the mixture-CAT scores were based on five or less items, 33% on six to nine items, and 18% on 10 items (the maximum allowed). The simulation analyses and the corresponding comparisons of A:B, A:C, and A:D (see Figs 1 and 2) revealed substantial bias in the latent variable model-predicted scores when sample heterogeneity was ignored. The first comparison made was to evaluate how well the mixture-CAT scores (i.e., the model predicted PRO scores) approximated the referent scores (comparison A:B in Fig 1). On the left of Fig 3A, the referent scores are plotted on the x-axis and compared with the mixture-CAT PRO scores on the y-axis. Their differences (i.e., the referent score minus the mixture-CAT score, or bias) are shown on the right of Fig 3A. It is shown that the mixture-CAT reproduced the referent scores nearly perfectly for all three classes. The reliability, based on the squared correlation with the referent scores, was .99. Table 3 shows the corresponding relative cumulative frequency distribution of the differences between the referent scores and the mixture-CAT scores. The largest overestimation is .23 SDs from the referent score, and the largest underestimation is .16 SDs; 5% of the scores are overestimated by ≥ .12 SDs and 5% are underestimated by ≥ .09 SDs.

**Table 3:** Cumulative Frequency Distributions of Difference Scores of Theta.

	Mixture CAT using LVMM parameters	Conventional IRT model (all items)	Conventional CAT
Relative cumulative frequency (%)	Difference score (A: B)	Difference score (A: C)	Difference score (A: D)
Minimum	-0.23		
5	-0.12	-0.52	-0.43
10	-0.07	-0.44	-0.37
25	-0.03	-0.27	-0.23
50	0.01	-0.06	-0.01
75	0.04	0.32	0.26
90	0.07	0.48	0.40
95	0.09	0.50	0.42
Maximum	0.16		

*Notes*. The difference scores are calculated by subtracting the model predicted PRO scores from the referent PRO scores.

To evaluate the bias that arose when sample heterogeneity was ignored, we compared the latent variable model-predicted PRO scores derived from steps C and D (see Fig 1) with the referent PRO scores (step A). In the A:C comparison (i.e., the referent scores versus the conventional IRT model predicted PRO scores), as shown in Fig 3B, we see substantial differences. The average reliability, based on the squared correlation of the referent scores, was .91. Despite good reliability, on average, it is noteworthy that 50% of the cases had conventional model predicted PRO scores that were at least ±0.30 SDs from their corresponding referent scores, and 20% of the scores differed by ±0.46, in the overall population. With respect to the specific latent classes, Fig 3B further depicts that the model predicted PRO scores of latent classes 1 and 2 were primarily overestimated, whereas the model predicted PRO scores were predominantly underestimated for latent class 3. The shape of the overall scatter shows that the bias was greater for relatively lower referent PRO scores, which were indicative of relatively less pain.

The comparisons in Fig 3C show the bias in the model-predicted PRO scores produced by the conventional CAT when sample heterogeneity was ignored, compared with the referent PRO scores (A:D). As is shown in Table 3, 50% of the cases had conventional CAT-predicted PRO scores that were at least ±0.24 from their referent scores, and 20% of the cases differed by ±0.40. The bias within classes was similar to that of the conventional model predicted PRO scores with latent classes 1 and 2 being primarily overestimated and latent class 3 being underestimated. Again, the bias was greater for lower referent PRO scores (i.e., relatively less pain).

## Discussion

Conventionally, CATs for the measurement of PROMs are based on a single set of measurement model parameters that are assumed to be applicable to all people in the population, irrespective of any differences among them. Our study demonstrates that ignoring heterogeneity in a population can result in biased model-predicted latent variable scores, including those produced by a CAT. In measuring pain using the CAT-5D-QOL, we found that the bias was greatest in people with relatively less pain (i.e., lower model predicted PRO scores). At this less severe end of the pain continuum, the level of pain tended to be overestimated for people in classes 1 and 2 (i.e., the people who, relative to class 3, were older, more likely to have been taking medications, more likely to have had osteoarthritis or another health condition, and more likely to have reported poor or fair health). Conversely, the level of pain tended to be underestimated for people in class 3. The implication of this bias is that the measurement of pain was relatively unbiased for people with severe pain and became increasingly biased when less pain was present.

The results also demonstrate that the bias in the conventional CAT (ignoring heterogeneity) was nearly equivalent to the bias observed in the conventional IRT model that included all of the 36 pain items. Importantly, relative to using all the items, the use of CAT did not introduce additional bias when heterogeneity in the sample was ignored. In other words, the observed bias is the result of using an incorrect measurement model (that assumes parameter invariance) for the computation of latent variable scores. We further found that bias can be reduced by computing scores based on the parameters of an IRT mixture model to accommodate heterogeneity. This involves using class-specific measurement model parameters and information about latent class membership to compute the PRO scores. Indeed, we found that the mixture CAT produced scores that closely approximated the referent scores that were used to generate the data in our simulation study.

An important benefit of a CAT is that reliable scores can be obtained with minimal burden to the respondent. Indeed, the results of the simulation study indicate that the CAT-predicted PRO scores very closely approximated the referent PRO scores. However, it is important to note that, like all latent variable model-based scores, this benefit is conditional on the extent to which the fundamental assumption of local independence holds true [10, 12]. As is aptly described in the Draper-Lindley-de Finetti (DLD) framework of measurement validation [15, 16], local independence exists when all dependencies among *items* and *persons* are accounted for by the measurement model. That is, generalizable measurement inferences require independence (or exchangeability) of both items and persons. This is particularly important when CATs, that involve administration of different items to different people, are used to obtain scores. The items must be exchangeable so that the scores of individuals who answered different questions are comparable on the same scale. The persons (or sampling units) must also be exchangeable (i.e., the items’ parameters must be invariant) so that the scores are comparable irrespective of any differences among individuals other than the characteristic being measured. Factor analysis and IRT have been widely used to establish measurement models that ensure the exchangeability of items. However, the exchangeability of persons (sample homogeneity) has been less commonly examined in PRO measurement validation studies. Our analyses suggest that bias in latent variable model-predicted PRO scores can be introduced when the lack of exchangeability of persons in heterogeneous populations is ignored. Most important, we found that this bias can be mitigated by using parameters from a latent variable mixture model (LVMM) to predict PRO scores that accommodate heterogeneity.

Despite these promising results, there are several limitations to keep in mind. First, the computation of predicted scores using LVMM requires that the posterior probability of latent class membership is known. In our study, the posterior probabilities were obtained by fitting a latent variable mixture model to the data. However, when the LVMM is applied to different people whose posterior probabilities are not known, one needs to rely on predictors of latent class membership. For example, it is possible to predict latent class membership using the variables in our latent class regression model (see Table 2). This is an area for further empirical research and simulation analysis. In particular, it is not known how accurate the prediction of latent class membership would need to be to predict accurate PRO scores. Second, the current analyses are based on parameters that were derived from particular items and a particular sample. Further simulation analysis is required to determine the extent to which different sizes and numbers of latent classes, and differences in entropy, may lead to different degrees of bias. We recommend replicating these analyses in larger and representative population-based samples. Third, simulation studies examining the impact of different fixed-length and variable-length stopping rules in relation to latent variable mixture modeling CATs are recommended [63, 67].

Finally, although the parameters used for the mixture CAT were estimated using original observed data, they were not re-estimated for each simulated dataset. Further research needs to be done to examine the accuracy of IRT mixture model parameter recovery [68]. However, this was not the purpose of the simulation study presented herein. Rather, we sought to examine whether a mixture CAT (using IRT mixture model parameters) would be more accurate in predicting PRO scores, relative to a CAT based on a conventional (one-class) IRT model, when applied to a heterogeneous sample. There are several challenges in the estimation of IRT mixture model parameters that need to be resolved [68]. For example, it is not feasible to test the invariance of each item individually because the latent classes become redefined every time a different item is examined. The current study focused specifically on examining differences in measurement model parameters when the model predicted PRO scores were scaled with a mean of zero and a variance of one within each of the classes. Further research is recommended to examine the ideal conditions for accurate parameter recovery.

Heterogeneity within a population could pose a significant challenge for model-based approaches to PRO measurement, including CATs. Ignoring such heterogeneity could result in biased PRO scores. This bias can be mitigated by using LVMM, including in the CAT context, to establish measurement models that accommodate heterogeneity in a population. Further research is needed to evaluate the implications of sample heterogeneity with respect to the use of CAT-predicted PRO scores in research and clinical decision making.

## Supporting Information

S1 Appendix Item parameters of the latent variable mixture model (3 classes). (PDF)
