# Supplementary material for: The Accuracy of Computerized Adaptive Testing in Heterogeneous Populations: A Mixture Item-Response Theory Analysis
Source: PLoS One. 2016 Mar 1;11(3):e0150563. doi: 10.1371/journal.pone.0150563 (PMC4773251; doi:10.1371/journal.pone.0150563)
Supplement: S1 Appendix — (PDF) [file pone.0150563.s001.pdf]

Supporting Information

S1 Appendix: Item parameters of the latent variable mixture model (3 classes)

	Class 1					Class 2					Class 3				
Item	λ *(SE)*	τ_1 *(SE)*	τ_2 *(SE)*	τ_3 *(SE)*	τ_4 *(SE)*	λ *(SE)*	τ_1 *(SE)*	τ_2 *(SE)*	τ_3 *(SE)*	τ_4 *(SE)*	λ *(SE)*	τ_1 *(SE)*	τ_2 *(SE)*	τ_3 *(SE)*	τ_4 *(SE)*
1	4.3	-5.0	0.6	5.3		4.3	-7.4	-0.4	4.2		5.3	-3.3	3.5	7.5	
	(.45)	(.54)	(.43)	(.59)		(.48)	(.79)	(.38)	(.52)		(.50)	(.49)	(.40)	(.61)	
*2**	4.1	-5.7	-2.2	0.2	4.2	4.0	-7.7	-3.4	-0.7	3.2	4.9	-3.9	0.9	3.0	6.7
	(.35)	(.55)	(.41)	(.40)	(.45)	(.40)	(.74)	(.46)	(.36)	(.43)	(.44)	(.49)	(.32)	(.34)	(.52)
3	4.5	-3.1	1.3	5.0		4.8	-1.4	2.0	5.9		6.3	1.7	5.3	8.4	
	(.47)	(.51)	(.40)	(.55)		(.47)	(.44)	(.40)	(.52)		(.53)	(.41)	(.45)	(.60)	
4	6.0	-5.4	1.2	4.8	9.1	6.1	-2.8	2.3	4.9	9.1	6.1	0.4	4.5	6.7	9.1
	(.38)	(.76)	(.51)	(.45)	(.42)	(.48)	(.60)	(.45)	(.44)	(.42)	(.42)	(.38)	(.35)	(.39)	(.42)
5	3.9	-2.8	1.5	4.0	6.8	3.4	-5.3	-0.1	2.2	5.9	3.9	3.1	5.4	7.1	9.6
	(.49)	(.47)	(.42)	(.54)	(.69)	(.43)	(.53)	(.29)	(.31)	(.54)	(.48)	(.45)	(.54)	(.67)	(.08)
6	4.6	-4.3	0.5	2.6	5.4	4.7	-2.9	0.7	2.9	5.8	5.3	0.1	3.1	4.6	6.5
	(.45)	(.49)	(.43)	(.44)	(.60)	(.55)	(.47)	(.38)	(.45)	(.67)	(.37)	(.34)	(.37)	(.42)	(.47)
7	3.8	-2.9	-0.2	3.4	7.0	3.5	-3.0	-0.7	2.4	5.7	3.3	0.9	2.5	4.3	6.8
	(.33)	(.38)	(.36)	(.41)	(.54)	(.32)	(.36)	(.33)	(.35)	(.42)	(.26)	(.23)	(.25)	(.29)	(.45)
8	3.1	-1.2	2.3	4.5	8.7	2.1	0.2	2.5	4.1	7.2	2.8	2.9	4.9	6.6	7.4
	(.34)	(.35)	(.37)	(.44)	(.91)	(.27)	(.21)	(.25)	(.31)	(.69)	(.32)	(.32)	(.43)	(.53)	(.64)
9	4.0	-2.0	2.4	5.1	8.3	3.7	0.7	3.4	5.2	8.4	4.7	3.3	5.7	7.4	9.2
	(.42)	(.47)	(.43)	(.51)	(.76)	(.38)	(.34)	(.38)	(.48)	(.85)	(.49)	(.45)	(.53)	(.64)	(.74)
10	3.4	0.5	2.4	4.9	7.8	2.2	2.9	4.3	6.5	7.7	4.0	5.0	6.6	8.1	10.0
	(.38)	(.37)	(.39)	(.51)	(.82)	(.35)	(.35)	(.42)	(.64)	(.26)	(.57)	(.56)	(.66)	(.83)	(.20)
11	5.0	-0.1	4.3	6.7	10.8	2.1	2.0	4.2	5.5	7.3	4.0	4.0	6.4	7.8	9.3
	(.55)	(.51)	(.61)	(.70)	(.02)	(.28)	(.28)	(.33)	(.43)	(.68)	(.41)	(.43)	(.51)	(.62)	(.77)
12	3.0	1.6	4.1	5.6	8.5	1.9	2.7	4.8	6.0	7.3	2.9	4.5	6.5	7.7	9.1
	(.34)	(.33)	(.42)	(.49)	(.84)	(.28)	(.30)	(.40)	(.59)	(.74)	(.39)	(.46)	(.56)	(.69)	(.03)
13	3.8	-0.9	3.0	4.8	7.7	3.0	1.4	3.4	5.3	7.8	3.9	3.3	5.5	7.2	8.4
	(.35)	(.39)	(.42)	(.50)	(.71)	(.34)	(.30)	(.35)	(.45)	(.66)	(.38)	(.37)	(.47)	(.57)	(.68)
14	3.1	0.7	2.9	5.1	7.0	2.0	3.1	4.6	6.6	36.9	3.3	4.8	6.3	7.8	8.9
	(.33)	(.35)	(.40)	(.48)	(.62)	(.35)	(.41)	(.46)	(.64)	(.00)	(.43)	(.52)	(.63)	(.71)	(.85)

Item	Class 1					Class 2					Class 3				
	λ (SE)	τ_1 (SE)	τ_2 (SE)	τ_3 (SE)	τ_4 (SE)	λ (SE)	τ_1 (SE)	τ_2 (SE)	τ_3 (SE)	τ_4 (SE)	λ (SE)	τ_1 (SE)	τ_2 (SE)	τ_3 (SE)	τ_4 (SE)
15	2.2 (.30)	2.2 (.33)	3.9 (.37)	6.3 (.55)	8.0 (.06)	2.1 (.56)	5.1 (.86)	6.6 (.03)	7.7 (.21)	36.8 (.00)	6.4 (.41)	12.1 (.53)	13.6 (.66)	16.3 (.08)	18.6 (.49)
16	3.1 (.41)	-4.1 (.51)	-0.5 (.31)	0.4 (.31)	2.2 (.41)	3.0 (.33)	-2.4 (.32)	-0.5 (.27)	0.7 (.30)	2.3 (.35)	5.1 (.47)	-0.2 (.34)	1.7 (.37)	2.4 (.39)	3.9 (.42)
17	3.0 (.32)	1.0 (.38)	3.3 (.41)	5.3 (.46)	8.1 (.73)	2.5 (.40)	4.0 (.45)	5.2 (.51)	7.4 (.81)	42.0 (.00)	2.7 (.40)	4.8 (.50)	6.5 (.57)	7.3 (.68)	8.4 (.98)
18	3.5 (.38)	-3.2 (.40)	1.4 (.45)	3.8 (.55)	6.9 (.72)	3.5 (.53)	-6.5 (.60)	-0.3 (.30)	2.1 (.36)	5.7 (.63)	3.8 (.35)	2.6 (.35)	5.3 (.42)	7.2 (.54)	8.8 (.79)
19	4.4 (.45)	-3.2 (.53)	1.8 (.46)	4.4 (.55)	7.8 (.75)	3.6 (.34)	-0.1 (.34)	2.4 (.34)	4.4 (.42)	7.7 (.85)	5.5 (.52)	3.3 (.51)	5.6 (.57)	7.3 (.64)	9.1 (.77)
20	2.5 (.28)	0.4 (.30)	3.1 (.36)	5.0 (.42)	7.7 (.85)	1.8 (.29)	2.4 (.30)	4.1 (.37)	5.6 (.59)	36.4 (.00)	3.6 (.69)	5.8 (.88)	7.4 (.99)	9.8 (.77)	36.1 (.00)
21	4.2 (.44)	-3.9 (.50)	1.0 (.42)	3.1 (.49)	6.2 (.71)	3.8 (.37)	-1.6 (.37)	1.4 (.32)	3.1 (.37)	6.3 (.51)	5.6 (.40)	1.1 (.37)	4.3 (.44)	6.0 (.51)	8.0 (.55)
22	3.3 (.34)	-3.4 (.41)	-0.7 (.32)	0.4 (.34)	2.4 (.39)	3.8 (.39)	-2.3 (.38)	0.1 (.33)	1.5 (.35)	3.7 (.44)	5.0 (.37)	0.8 (.33)	2.3 (.34)	3.0 (.36)	4.5 (.41)
23	2.3 (.27)	0.5 (.24)	2.4 (.28)	4.5 (.36)	7.4 (.03)	2.0 (.21)	2.4 (.28)	3.4 (.30)	5.6 (.56)	33.6 (.00)	2.1 (.17)	6.5 (.92)	7.4 (.94)	10.0 (.59)	35.3 (.00)
24	1.8 (.22)	-4.2 (.53)	0.2 (.43)	4.5 (.49)	8.4 (.73)	1.6 (.28)	-0.9 (.40)	1.2 (.40)	4.5 (.43)	7.4 (.64)	3.5 (.68)	2.3 (.45)	4.4 (.46)	7.1 (.59)	9.5 (.75)
25	4.4 (.39)	-2.8 (.31)	-0.4 (.26)	1.6 (.29)	3.8 (.37)	4.3 (.40)	-1.7 (.29)	0.1 (.27)	1.6 (.29)	3.8 (.36)	5.8 (.49)	0.3 (.28)	1.7 (.31)	2.9 (.33)	4.8 (.37)
26	2.5 (.25)	-4.2 (.45)	0.6 (.42)	3.2 (.45)	6.7 (.61)	2.9 (.29)	-2.8 (.44)	1.0 (.42)	3.0 (.43)	6.0 (.52)	4.0 (.31)	0.6 (.37)	3.4 (.39)	5.3 (.44)	8.3 (.56)
27	4.2 (.39)	-4.2 (.42)	-0.1 (.34)	1.9 (.38)	5.0 (.51)	4.6 (.43)	-3.8 (.33)	-0.5 (.25)	1.2 (.28)	3.6 (.35)	5.5 (.39)	-0.5 (.23)	1.9 (.23)	3.4 (.28)	5.5 (.35)
28	3.6 (.34)	-2.6 (.37)	0.0 (.36)	3.3 (.46)	6.6 (.59)	3.0 (.30)	-4.1 (.39)	-1.3 (.27)	1.8 (.25)	5.1 (.45)	3.4 (.22)	3.0 (.41)	5.1 (.46)	7.7 (.70)	11.4 (.74)
29	3.0 (.35)	-3.1 (.38)	-0.4 (.30)	2.7 (.40)	5.2 (.51)	2.6 (.35)	-3.9 (.31)	-1.3 (.21)	1.2 (.20)	3.6 (.30)	3.8 (.39)	1.3 (.24)	3.0 (.26)	5.2 (.37)	7.5 (.80)
30	2.7 (.30)	-4.9 (.61)	1.3 (.58)	5.1 (.69)	11.5 (.17)	2.1 (.26)	-2.1 (.56)	2.4 (.53)	5.1 (.63)	9.4 (.93)	2.7 (.26)	2.1 (.42)	5.3 (.47)	7.4 (.59)	10.2 (.78)

Item	Class 1					Class 2					Class 3				
	λ (SE)	τ_1 (SE)	τ_2 (SE)	τ_3 (SE)	τ_4 (SE)	λ (SE)	τ_1 (SE)	τ_2 (SE)	τ_3 (SE)	τ_4 (SE)	λ (SE)	τ_1 (SE)	τ_2 (SE)	τ_3 (SE)	τ_4 (SE)
31	5.9 (.57)	-5.5 (.55)	-1.4 (.43)	2.6 (.46)	7.2 (.68)	6.0 (.58)	-8.1 (.13)	-1.3 (.41)	2.1 (.41)	7.0 (.66)	6.2 (.49)	-3.1 (.43)	1.8 (.31)	5.1 (.41)	8.2 (.59)
32	4.5 (.43)	1.2 (.30)	2.6 (.32)	4.2 (.38)	5.9 (.58)	4.7 (.52)	4.0 (.51)	5.4 (.56)	6.8 (.80)	7.7 (.30)	4.9 (.41)	4.9 (.54)	5.7 (.57)	6.6 (.64)	7.1 (.66)
33	2.3 (.25)	0.0 (.24)	1.8 (.26)	3.9 (.35)	7.7 (.03)	2.2 (.39)	2.0 (.29)	2.9 (.30)	5.2 (.46)	8.3 (.45)	3.1 (.45)	4.3 (.51)	5.9 (.55)	7.2 (.67)	8.5 (.97)
34	2.1 (.23)	-0.2 (.29)	1.7 (.31)	4.2 (.40)	7.4 (.68)	2.3 (.31)	0.4 (.27)	1.7 (.29)	4.0 (.39)	7.8 (.63)	3.3 (.47)	3.4 (.37)	4.6 (.39)	6.3 (.52)	9.0 (.80)
35	2.6 (.28)	-4.2 (.60)	6.4 (.94)			2.8 (.35)	-5.4 (.60)	4.8 (.71)			3.2 (.34)	-1.4 (.34)	6.5 (.61)		
36	4.6 (.65)	-5.8 (.65)	-0.9 (.25)	0.5 (.26)	2.3 (.31)	4.0 (.57)	-4.9 (.40)	-1.0 (.21)	0.4 (.20)	1.5 (.22)	3.9 (.42)	-2.9 (.22)	0.7 (.16)	1.7 (.17)	2.4 (.21)

Notes. The parameters are of a mixture graded response model as specified in the MPlus [39] software where the cumulative probability (P_{ij}) of an item *i* response at or above category *j* is expressed as follows (see Sawatzky et al. [34] for details about model specification):

$$P_{ij}\left(Y \geq j \mid \theta\right) = \frac{\exp(-\tau_{ij} + \lambda_i \theta)}{1 + \exp(-\tau_{ij} + \lambda_i \theta)},$$

where τ_{ij} denotes the thresholds between the categories of item *i*, and λ_i denotes the factor loading for item *i*. The following transformation can be applied to convert the Mplus thresholds (τ) and factor loadings (λ) into the difficulty (β) and discrimination (α) parameters of the grade response model:

$$\beta_{ij} = \frac{\tau_{ij}}{\lambda_i} \text{ and } \alpha_i = \lambda_i .$$

* Item 2 is the only item with five thresholds. The λ and τ estimates for the fifth threshold are 7.4(.62), 6.7(.57) and 10.0(.78) for classes 1, 2 and 3.
